# An investigation on the cyclic temperature-dependent performance behaviors of ultrabright air-stable QLEDs

**DOI:** 10.1038/s41598-023-39952-3

**Published:** 2023-08-05

**Authors:** Saeedeh Mokarian Zanjani, Sadra Sadeghi, Afshin Shahalizad, Majid Pahlevani

**Affiliations:** 1 Department of Electrical and Computer Engineering, Queen’s University, Kingston, ON K7L 3N6 Canada; 2 Genoptic LED Inc., Calgary, AB T2C 5C3 Canada

**Keywords:** Condensed-matter physics, Materials for devices, Materials for optics, Nanoscale materials, Structural materials

## Abstract

The aerobic and thermal stability of quantum-dot light-emitting diodes (QLEDs) is an important factor for the practical applications of these devices under harsh environmental conditions. We demonstrate all-solution-processed amber QLEDs with an external quantum efficiency (EQE) of > 14% with almost negligible efficiency roll-off (droop) and a peak brightness of > 600,000 cd/m^2^, unprecedented for QLEDs fabricated under ambient air conditions. We investigate the device efficiency and brightness level at a temperature range between − 10 and 85 °C in a 5-step cooling/heating cycle. We conducted the experiments at brightness levels higher than 10,000 cd/m^2^, required for outdoor lighting applications. Our device performance proves thermal stability, with minimal standard deviation in the performance parameters. Interestingly, the device efficiency parameters recover to the initial values upon returning to room temperature. The variations in the performance are correlated with the modification of charge transport characteristics and induced radiative/non-radiative exciton relaxation dynamics at different temperatures. Being complementary to previous studies on the subject, the present work is expected to shed light on the potential feasibility of realizing aerobic-stable ultrabright droop-free QLEDs and encourage further research for solid-state lighting applications.

## Introduction

Quantum-dot light-emitting diodes (QLEDs) have sparked a significant amount of attention from both academia and industry, owing to their exceptional optoelectronic properties, making them suitable for various electronic devices^1–7^. For example, external quantum efficiency (EQE)^8–11^, brightness level^12,13^, and operational lifetime^14–17^ of QLEDs have now reached the standards for commercial displays^18^. On the other hand, taking advantage of luminescent colloidal quantum-dots (QDs) with well-engineered graded multi-shell configurations, negligible EQE roll-off (droop) of QLEDs at high brightness levels^19^ may allow for their applications in outdoor lighting, projection displays, and phototherapy^19–21^. However, due to issues such as operational stability, shelf stability, and efficiency roll-off, reliable high-brightness, QLEDs suitable for solid-state lighting systems are still far away from commercialization^21^.

In that context, thermal stability at high brightness levels with preserved efficiency under harsh environmental conditions (i.e., extreme temperatures and high humidity levels) is a key factor for outdoor LED lighting systems. Device protection against oxygen and moisture at high humidity levels (e.g., 85 RH) can be ensured by employing advanced thin-film encapsulation methods^22^, but the thermal stability of an LED system depends on both intrinsic and modified charge transport characteristics in the functional charge transport layers^23^ as well as exciton relaxation dynamics in the emissive layer (EML). Several studies have been published previously on the temperature-dependent electroluminescence (EL) performance of organic LEDs (OLEDs)^24–26^, perovskite LEDs^27,28^, and QLEDs^29–31^. As the focus of the present work, for example, M. Zhang et al. investigated both photoluminescence (PL) and EL performance of their red QLEDs within a 120–300 K temperature range but did not carry out their experiments at temperatures higher than room temperature (RT)^31^. The authors reported enhancement of the current density and reduction of turn-on voltage as the temperature was increased to RT from sub-zero temperatures. In a study by J. Yun et al., the authors explored the current density vs. voltage (J-V) behavior of their inverted colloidal Cd-based QLEDs at 100–400 K but did not report efficiency parameters^29^. Biswas et al. implemented a heat-assisted method for improving the EQE and current efficiency (LE) of their colloidal CuInS-based yellow QLEDs^30^. These authors observed that by increasing the substrate temperature during the sputtering of ZnO electron transport layer (ETL), the charge injection of their devices improved, which led to an efficiency enhancement. Recently, Sue et al. proposed that the root cause of up-conversion EL (i.e., sub-bandgap turn-on EL), typically observed in QLEDs, is due to thermally-assisted charge injection by exposing their devices to a broad temperature range. However, these authors performed their experiments within a small voltage range (around the turn-on voltage) and apparently did not investigate the temperature-dependency at high brightness levels^32^. Additionally, one should note that, even without exposure to external extreme temperatures, the operating temperature of QLEDs at high brightness levels typically exceeds RT, due to the high current passing through the device, causing Joule heating^13,33,34^. Therefore, proper thermal management is crucial to long-term operational stability of ultrabright QLEDs. Moreover, the effects of temperature, electric field, and positive aging on the device performance were studied before. In the case of QLED devices, in situ, interfacial reactions can enhance device efficiency by reducing charge leakage. In addition, resistive switching, achieved through electric field application using oxide ETL in QLED, leads to the movement of oxygen vacancies and the formation of conductive filaments, which induces positive aging in QLEDs^23^. However, high electric fields can accelerate the wear and degradation of QLED materials, potentially impacting device performance and lifespan. Similarly, elevated temperatures can affect QLED efficiency, stability, and lifespan, leading to increased power consumption, material degradation, reduced luminosity, and potential device failure. While higher temperatures and electric fields may improve carrier transport and brightness, they can decrease the operational lifetime of the device. In a study by C. Lee et.al, increasing the annealing temperature up to 200°C was found to enhance QLED performance due to reinforced hole injection. However, further temperature increases resulted in reduced efficiencies due to degradation of hole injection and increased electron injection rates, leading to charge accumulation^35^. Z. Chen et al., investigated the effect of HTL and positive aging on the performance and life span of the QLED device^36^. The use of a desiccant improved the stability of the hole transport layer (HTL) in QLEDs, suppressing positive aging. Conversely, devices without a desiccant degraded faster but showed positive aging. This indicates a trade-off between positive aging and HTL stability. Overall, positive aging in QLEDs can be achieved through interfacial reactions, resistive switching, and controlled operating conditions, while high temperatures and electric fields could potentially have detrimental effects on device performance and operational lifetime.

**Figure 1:**
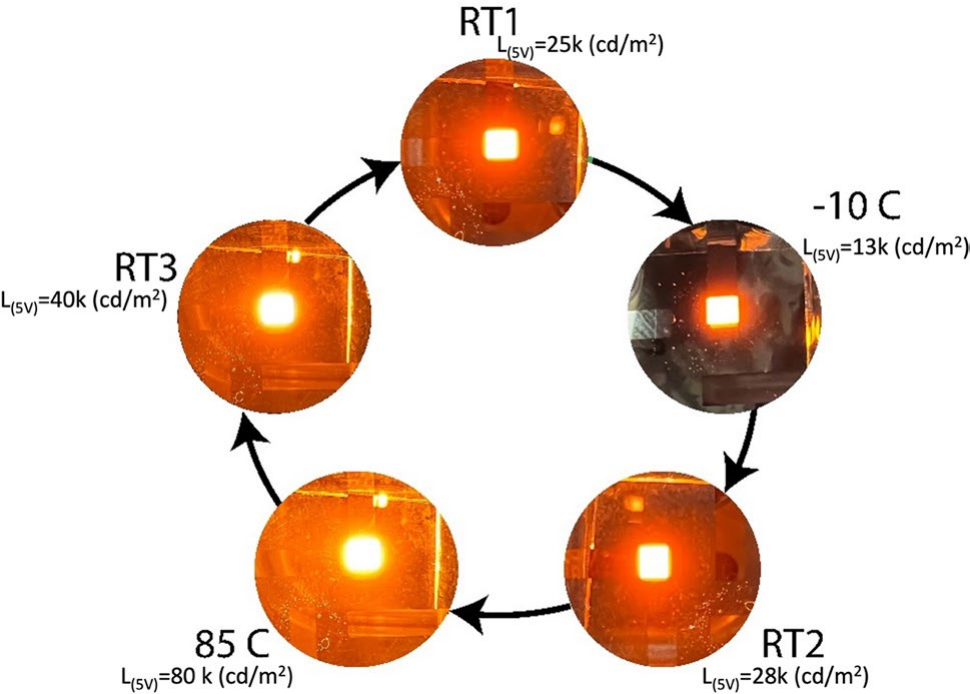
Photographs of an amber bottom-emitting QLED operated under multiple heating/cooling cycles.

Herein, we report on solution-processed air-stable amber QLEDs (EL peak at 600 nm) with extremely narrow emission linewidth and study their thermally-induced optoelectronic behaviors. Our devices show a high maximum EQE of over 14% and a maximum brightness of 624,000 cd/m^2^ at 12 V, unprecedented for QLEDs fabricated under ambient-air conditions (see, e.g., Ref.^37^)^38^. We have experimentally investigated the temperature-dependent efficiency parameters and brightness by stressing the devices under multiple cooling/heating cycles within a wide temperature range from − 10 to 85 °C. Unlike some results previously published in the literature^29^, no device failure was observed after heating the devices up to 85 °C. Specifically, we operated our devices in a 5-step thermal cycle by starting from RT1, cooling down to − 10 °C, returning to RT2, heating up to 85 °C, and finally returning to RT3, sequentially. Compared to RT, results reveal a slight increase in the efficiency at − 10 °C and a slight reduction of efficiency at 85 °C (Fig. 1). However, the overall device efficiency remains almost unchanged within the experimental range of uncertainty. The biggest change is observed in terms of the brightness level, which is significantly affected by the applied temperature. The charge transport characteristics were also modified at different temperatures, increasing and reducing the turn-on voltage at low and high temperatures, respectively. Furthermore, the PL spectra of the QDs thin films were recorded at different temperatures to be able to distinguish between optical and electrical effects. Particularly, a temperature-dependent spectral shift in the emission peak and intensity was distinctly observed in both PL and EL. The present work provides insights into the feasibility of realizing air-stable, thermally-stable, and efficient QLEDs potentially suitable for practical outdoor lighting and display applications.

## Results and discussions

### Materials and QLED device characterization

**Figure 2:**
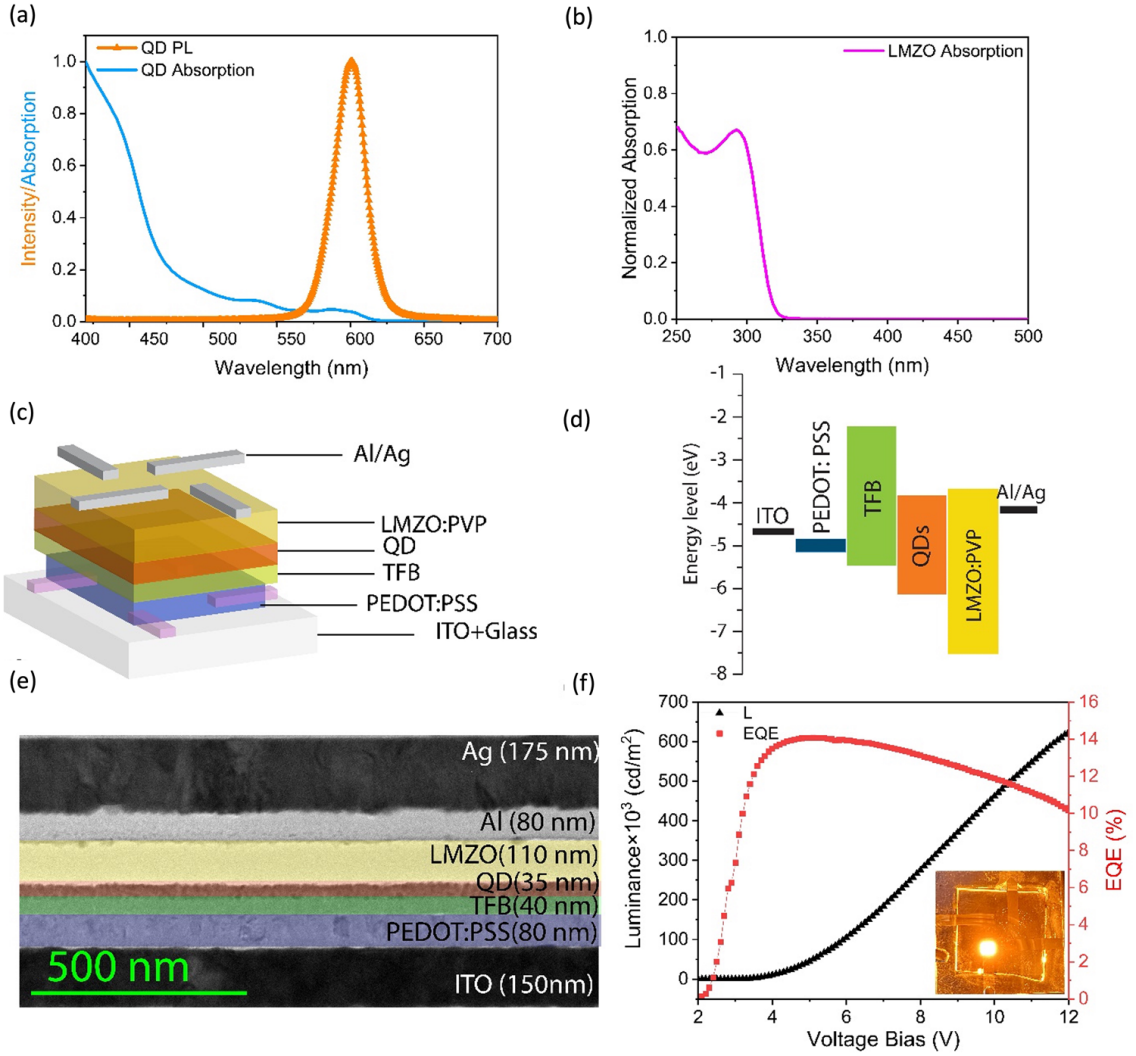
**(a)** Absorption and PL spectra of the QDs, **(b)** absorption spectrum of LMZO in solution **(c)** layer-stack of the QLED **(d),** energy level alignments of the layer-stack **(e),** cross-sectional TEM image of the QLED structure **(f),** and performance parameters. The inset in (**g**) shows the photograph of a QLED device operated at 12 V.

We synthesized ZnCdSe/ZnSe/ZnSeS/ZnS amber QDs as the EML and 10%Li :10%Mg:ZnO (LMZO) nanoparticles (NPs) as the ETL in the QLED structure. Since the focus of the present work is primarily on temperature effects, details of the QDs synthesis with full characterizations will be published elsewhere. The characterizations of QDs and LMZO NPs, the device structure, and the QLED performance are given in Fig. 2.

Figure 2a shows the PL and absorption spectrum of the QDs, where the PL peak is centered at 599 nm with the full width at half maximum (FWHM) of 21 nm. The QDs solution photoluminescence quantum yield (PLQY) measurements shows an average of 97 ± 2%, attributed to the strong confinement of electrons and holes inside the ZnCdSe core, which leads to the efficient core emission instead of the emission from the other shell materials. Additionally, the formation of the ultra-thick ZnSe/ZnSeS/ZnS shell (14 monolayers) on the core QDs effectively suppresses the Förster resonance energy transfer (FRET) between neighboring QDs in the film, leading to a high film PLQY value of 70%. The absorption spectrum obtained from UV–Vis spectroscopy shows the first excitonic peak position at 597 nm for the QDs (blue curve) and 294 nm for LMZO NPs (pink curve) in Fig. 2a and b, respectively. The LMZO bandgap is calculated to be 3.9 eV, based on the method used in reference^39^. The schematic of the spin-coated QLED device, energy band alignment, and the cross-sectional TEM image of the layer stack are shown in Fig. 2c–e.

The layers were spin coated on patterned ITO/glass substrates with a sheet resistance of 15–20 Ohm/sq. Poly(ethylene dioxythiophene): polystyrene sulfonate (PEDOT: PSS) with the thickness of 80 nm was used as the hole injection layer (HIL). A 40 nm-thick Poly[(9,9-dioctylfluorenyl-2,7-diyl)-co-(4,4’-(N-(p-butylphenyl)) diphenylamine)] (TFB) was used as the HTL. The QD layer with the thickness of 35 nm, and a 110 nm-thick LMZO were respectively the EML and ETL in the device structure. Finally, 80 nm of aluminum (Al) and 175 nm of silver (Ag) were deposited by thermal evaporation to serve as cathode. A UV-curable epoxy resin together with glass coverslips were used for the encapsulation before characterizing the devices in air. Except for the cathode, all the film spin coating process were carried out in the open air.

### Temperature-dependent PL and EL characteristics

**Figure 3:**
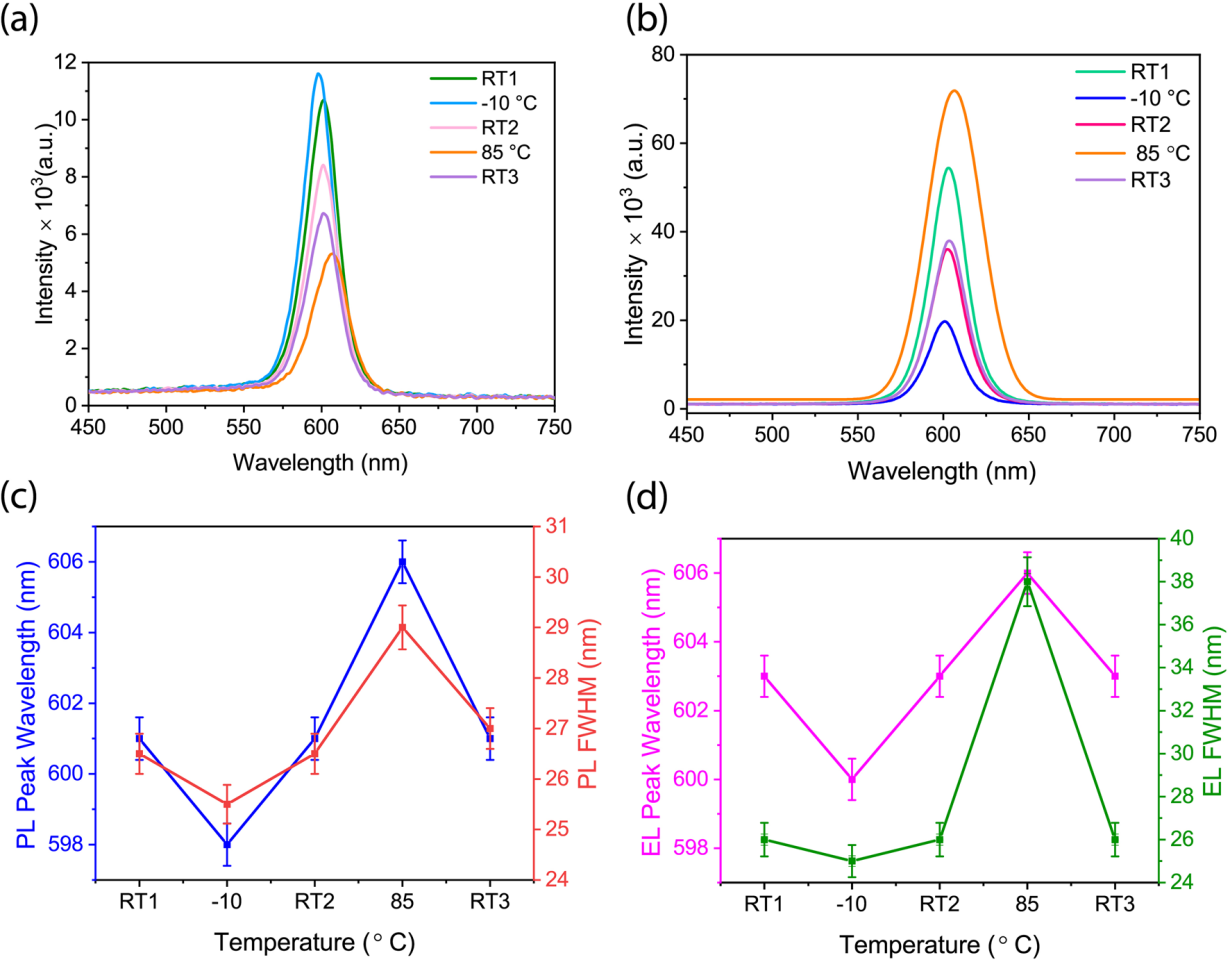
Temperature-dependence of **(a)** PL of the QDs film, **(b)** EL of the QLEDs recorded at a fixed driving voltage of 5V, **(c)** PL and **(d)** EL peak full width at half maximum (FWHM) and spectral position at − 10 to 85 °C.

In this section, first, we discuss the effect of cooling/heating cycles on the PL and EL characteristics of the QDs film and QLEDs, respectively. Figure 3a shows the PL spectra of the QDs films (excited at 365 nm) at different temperatures. According to Fig. 3a and c, the PL peak position undergoes a blue-shift at − 10 °C. Specifically, the PL peak shifts from 601 nm at RT1 to 598 nm and the spectral linewidth becomes narrower at − 10 °C (from 26.5 at RT1 nm to 25.4 nm), and then it retrieves to 601 nm when the film equilibrates to RT2. The origin of such a blue-shift in the PL peak has been previously found to be the bandgap expansion of QDs at low temperatures^31,40–42^. An opposite effect is also observed when the temperature rises to 85 °C, where the PL peak position undergoes a red-shift to 606 nm and the linewidth broadens (from 26.4 nm at RT2 to 29.3 nm), which are respectively ascribed to bandgap shrinkage and enhanced carrier-phonon scattering in QDs nanocrystals (i.e., the ZnCdSe core in our case) and subsequent defect emission at elevated temperatures^43–46^. As observed after the third cooling cycle, the peak position reversibly returns to 601 nm when the film equilibrates back to RT3, but the linewidth (27 nm) remains slightly wider than that at RT2.

On the other hand, the PL intensity enhances at − 10 °C, which can be attributed to the suppression of the non-radiative recombination channels through partial elimination of the temporary trap states formed due to the ion-exchange process^33^. Additionally, the probability of carrier localization for radiative recombination is higher at lower temperatures. In contrast, the carrier trapping (and subsequent non-radiative recombination) is expected to enhance at elevated temperatures due to formation of permanent and temporary trap states. This is clearly seen in the declined PL intensity at 85 °C. The partial recovery of the PL intensity upon cooling back to RT3 indicates relaxation of temporary trap states. The irreversible portion of the PL intensity can also be explained by emission quenching due to the formation of permanent trap states at elevated temperatures^43^. It is known that detachment of caping ligands in QDs solutions at elevated temperatures can contribute to formation of surface trap states^47^. However, in our case, since the 1-Octanethiol (OT) surface ligands are strongly bound to the QDs surface and considering that the measurements were done with solid-state films, it is unlikely that OT ligand detachment could cause significant exciton-quenching due to surface traps. It would not also be expected that the elevated temperature (85 °C) in our experiments can be sufficient for detaching the capping ligands because studies have shown that an onset temperature of around 100 °C would be needed^33^. Therefore, it may be expected that such temperature-induced trap states, which are casing the irreversible quenching, form in the shells or at the interfaces in the QD composition.

Figure 3b and d show the temperature-dependent EL spectral behaviors of an amber QLED device recorded at a fixed driving voltage. A similar spectral shift trend is also observed in the EL behavior during the cooling/heating cycles under the same experimental conditions. However, the changes in the emission intensity and spectral linewidth are found to be distinctly different. Specifically, the EL spectrum measured initially at RT1 (603 nm) shows a small 2 nm red-shift compared to the PL peak, but the linewidth remains the same. A blue-shift to 600 nm with a linewidth of 25.4 nm (similar to the PL) is observed when the device was cooled down to − 10 °C and then retrieves when equilibrating back to RT2. Zhang et al. ascribed such a spectral blue-shift and linewidth narrowing to carrier relaxation into lower energy localized states, and the change in charge-recombination dynamics at reduced temperatures^31^. The EL peak also red-shifts to 606 nm and a much broader linewidth of 37.4 nm is observed compared to the PL at 85 °C. Contrary to the PL behavior, however, the EL intensity declines at − 10 °C and is boosted significantly at 85 °C. After heating and cooling back to RT3, similar to the PL behavior, the EL intensity retrieves partially but not fully to their initial levels, likely due to the trap states discussed earlier.

**Figure 4:**
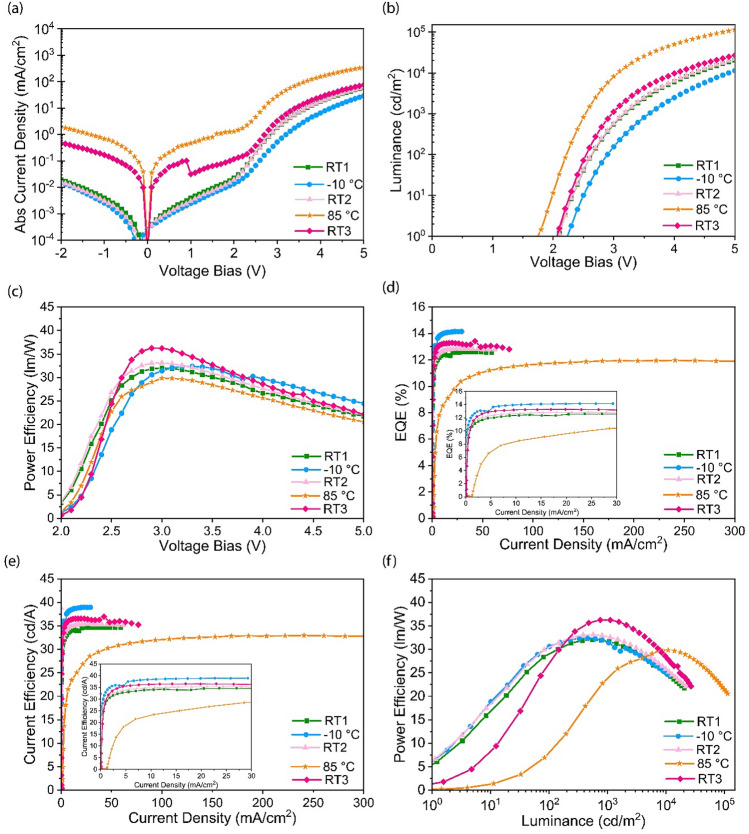
Amber QLED device performance at reduced and elevated temperature cycles. **(a)** Current density (J) **(b)** Brightness (L) and **(c)** Power efficiency (PE) versus voltage. **(d)** EQE_max_ vs. current density (J) **(e)** Current efficiency (LE) vs. current density (J) **(f)** Power efficiency (PE) vs. brightness (L). The insets of Fig. 3d and 3e show the magnified x- axis to better show the current density values at T < 85 °C.

The opposite trends observed in the PL and EL behaviors reflects that temperature-dependent charge injection (into the QDs) and charge transport modifications have a more impact than the effect of temperature at the QD level. For instance, it has been previously reported that the trapped electrons at elevated temperatures are released from the trap states and the charge transport and radiative recombination are enhanced^31^. Chen and coworkers^32^ reported from the results of their transient EL measurements, the devices were turned-on more rapidly due to enhanced hole injection, at elevated temperatures. The improved hole injection is due to the thermal energy, which facilitates overcoming the energy barrier and promotes the hopping transport of the holes^48^. So the improved charge transport at elevated temperatures clearly explains the higher current density and luminance of the device, while at lower temperatures a reverse mechanism occurs. To elaborate the observed effects, as discussed in the following, we investigated the variations in the temperature-dependent efficiency parameters, brightness level, and J-V behaviors in our QLEDs (Fig. 4 and Table 1).

**Table 1:** QLED device performance parameters at different temperatures. The voltage was swept up to 5 V.

T [°C]	RT1	− 10 °C	RT2	85	RT3
V_on_ [V]	2.1	2.3	2.1	1.8	2.1
EQE_max_ [%]	12.6	14.2	12.8	12.0	13.4
L_max_ × 10^3^ [cd/m^2^]	20.4	11.4	21.7	113.6	27.0
PE_max_ [lm/W]	32.0	32.4	33.1	29.8	36.2
LE_max_ [cd/A]	34.6	38.9	35.3	32.7	37.0
|J|_@5V_ [mA/cm^2^]	59.0	29.3	61.9	347.4	76.5

The initial brightness level at RT1 was recorded to be > 20,000 cd/cm^2^ at 5 V. Temperature-dependent EL studies have not been reported at such a high brightness level in literature. Figure 4a shows the J-V plots of the QLED at various temperatures. When the device is first cooled down to − 10°C from RT1, the current density declines, but when it equilibrates back to RT2, the current density retrieves almost to its initial value at RT1. In contrast, the current density values of the device shows that the charge transport properties are reinforced at the elevated temperature of 85°C. Owing to thermally-assisted charge injection at elevated temperatures^28^, the turn-on voltage (V_on_) reduces from 2.1 V at RT to 1.8 V at 85 °C. The V_on_ was also higher (2.3 V) at − 10°C, due to a reverse effect. In addition to thermally-assisted charge injection, given that the efficiency parameters drop slightly and considering that the brightness increases substantially at 85°C (Fig. 4b), the dramatically increased current density may also be correlated with increased leakage current within the voltage range in our experiments. Specifically, even though the current density at RT3 is lower than that at 85°C, it is still higher compared to its initial value at RT1. Given that the efficiency parameters do not completely return to the values at RT2, this may be due to any plausible minimal physical damage (due to the leakage current) occurring to the polymer HTL in the device structure. Such a physical damage at the HTL/QDs interface has been reported to be one of the main reasons for QLED degradations at high brightness/current density levels^49^. Another reason could be the thermal fluctuations in the thermal pad and the slight deviation of the temperature from RT2*.* Nevertheless, in our case, the likely damage to the HTL does not seem to be severe because the efficiency parameters return almost to their initial values at RT after the cooling/heating cycles, indicating thermal stability of the devices within the temperature range. Table 1 summarizes the QLED performance parameters operated in a thermal cycle.

The maximum power efficiency (PE_max_) shown in Fig. 4c and f are also found to be the lower at 85 °C compared to those recorded at RT, due to the increased turn-on voltage. On the other hand, the maximum current efficiency (LE_max_) is higher for the QLED operated at − 10 °C (Fig. 4e), which is in direct contrast with the previously reported results from Zhang et al.^31^. In addition, the EQE_max_ value is found to be higher at − 10 °C (Fig. 4d), which may be explained by the expected suppressed non-radiative recombination channels at reduced temperatures. One should note that the different lengths of the curves in Fig. 4d, and e at various temperatures originate from the variations in the maximum current density values at 5 V (the x-axis).

The change in temperature is also expected to affect the Fermi–Dirac distribution of band-edge carriers in QDs, and lead to a change of carrier behaviors and emission performance. At low temperatures near absolute zero, the Fermi–Dirac distribution function resembles a step function, with each energy level accommodating at most one electron. With increasing temperature, thermal broadening occurs, causing the Fermi–Dirac distribution to become smeared out and lose its step-like behavior. This results in an increased number of carriers in the band-edge states of QDs. In QLEDs, higher temperatures accelerate charge recombination rates due to increased kinetic energy of carriers. Thermal energy enables carriers to overcome energy barriers more easily, facilitating carrier recombination. Consequently, elevated temperatures can enhance charge recombination, impacting QLED efficiency and performance. On the other hand, at low temperatures, injected or generated electrons and holes in CdSe core–shell quantum dots are confined within the inner CdSe core, resulting in direct recombination for photon emission. As the temperature rises, more trapped carriers, particularly electrons, are thermally activated and released from surface trap sites of the outer ZnS shell. These delocalized carriers then enter the inner CdSe core, which subsequently undergo radiative recombination with confined holes. This thermally-assisted recombination benefits from elevated temperature by promoting carrier movement and recombination with holes^50^.

The Fermi–Dirac distribution in CdSe core–shell QDs is influenced by factors such as size, shape, composition, and surface properties. The interaction between core and shell materials also affects the energy level alignments and electronic properties. Density Functional Theory (DFT) calculations are necessary for a better understanding of the electronic distribution in core/multi-shell structures of QDs.

### Reproducibility and consistency of the QLED EL performance

**Figure 5:**
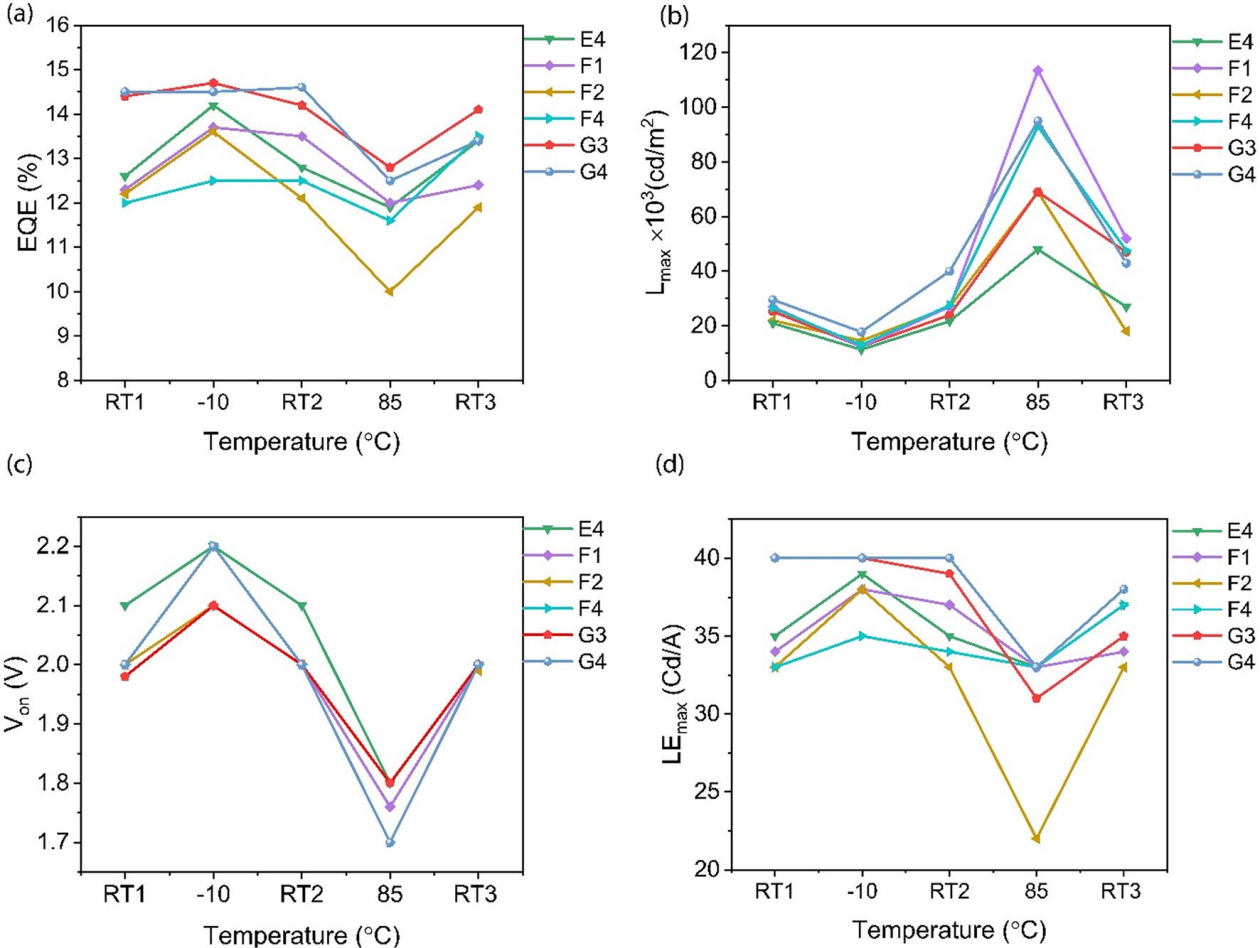
Effect of temperature on (**a)** EQE_max_, (**b)** L_max_, (**c)** V_on_, and **(d)** LE_max_ of the six structurally identical QLEDs.

The operation of QLEDs in cooling/heating cycles was repeated for different devices and the results were found to be fairly consistent. Figure 5 displays the trend of EQE_max_, L_max_, V_on_, and LE_max_ at different temperatures for six QLED devices which were structurally identical. According to Fig. 5a, the EQE_max_ first increases at − 10 °C, then it either remains unchanged, or reduces to near its initial value at RT2. Figure 5a, c, and d show that by increasing the temperature, EQE_max_, V_on_, and LE_max_ decrease, while the L_max_ value increases (Fig. 5b). The slight variations in the device performance parameters at each temperature arise from the thermal fluctuations in the Peltier plate during the measurements. Table 2 shows the average EQE_max_, V_on_, L_max_ and LE_max_ for six QLED devices with the standard deviations included at each temperature. These results show the consistency and reproducibility of our findings.

**Table 2:** Averaged efficiency parameters at different temperatures for six structurally identical devices.

T [ °C]	EQE_max_ [%]	L_max_ × 10^3^ [cd/m^2^] @5v	V_on_ [V]	LE_max_ [cd/A]
RT1	13.20 ± 1.24	24.81 ± 3.01	2.00 ± 0.03	36.17 ± 3.53
− 10	14.00 ± 1.15	13.11 ± 2.81	2.20 ± 0.05	38.17 ± 2.96
RT2	13.40 ± 1.16	28.20 ± 5.20	2.00 ± 0.04	36.50 ± 3.20
80	12.00 ± 0.90	79.44 ± 25.41	1.80 ± 0.05	31.33 ± 3.90
RT3	13.10 ± 0.88	41.63 ± 11.55	2.00 ± 0.03	35.17 ± 2.17

Importantly, none of the devices failed under multiple thermal cycling. Moreover, the EQE_max_ and V_on_ in most of the devices are retrieved to near their initial values. An efficiency enhancement at − 10 °C and a drop at 85 °C is observed while it recovers to its initial value after it cools down to RT3. In addition, L_max_ is improved at RT3 compared to L_max_ at RT1. Although the L_max_ drops at − 10 °C, it recovers when the device reaches the equilibrium with the ambient. V_on_ shows a completely elastic behavior, such that it increases from 2 V to 2.2 V at − 10 °C (reduction of carrier mobility) and recovers to 2 V at RT2. Followed by heating the device to 85 °C, the turn-on voltage reduces to − 1.8 V (enhanced carrier mobility at high T) and it recovers to 2 V at RT3. Finally, maximum current efficiency/luminance efficiency (LE_max_) values are shown in the fourth column of Table 2. According to the LE_max_ = L_max_/J, since the current density has its lowest value at − 10 °C, the current efficiency was the highest, 38 cd/A, when the temperature decreased. On the other hand, due to the maximum of current density at the elevated temperatures, the LE (L/J) at 85 °C has its minimum value. The current efficiency values almost recover to their initial value of 36 cd/A (RT1), at each equilibration with the ambient temperature (RT2, RT3).

**Figure 6:**
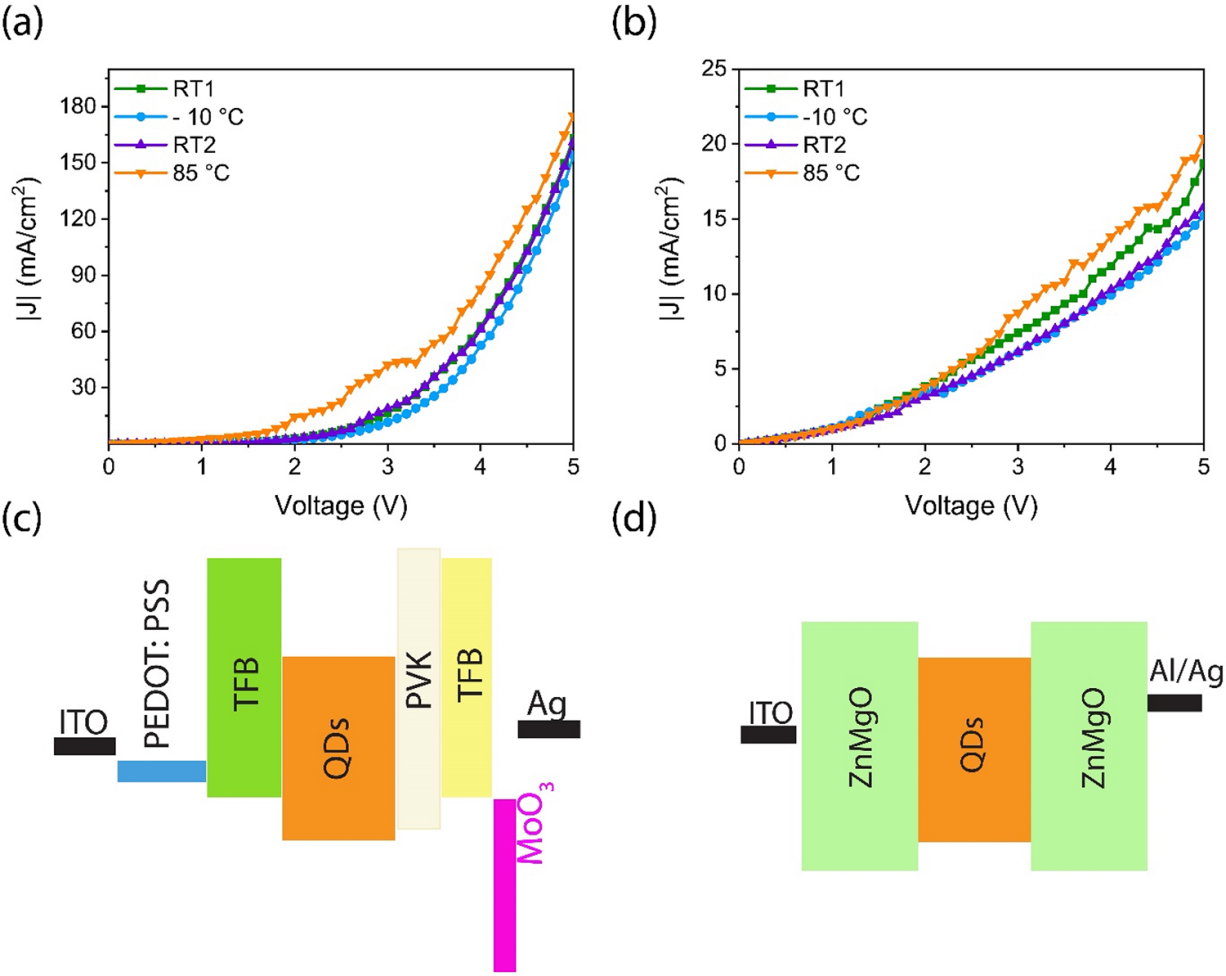
Effect of the temperature on the J-V curves of the **(a)** HOD, **(b)** EOD, **(c)** the schematic layer stack of HOD, and **(d)** EOD.

To better understand the effect of the temperature on the charge transport properties of the QLED devices, unipolar hole-only and electron-only devices (HOD and EOD) were fabricated, and their J-V curves were investigated in the cooling-heating cycles. Figure 6a and 6b display that the current density significantly increases at 85 °C in both EOD and HOD. Rise in the temperature, facilitates carrier transport and increases the current density. Figure 6c–d schematically show the HOD and EOD device structures, respectively.

## Conclusion

We have investigated the performance stability of our air-fabricated spin-coated amber-emitting QLED devices under thermal stress. These QLEDs exhibit an external quantum efficiency (EQE) exceeding 14%, with almost negligible efficiency roll-off (droop), and an unprecedented peak brightness of over 600,000 cd/m^2^. These results were achieved under ambient air conditions, a significant advancement in QLED technology. We investigate the device efficiency and brightness performance across a wide temperature range (− 10 to 85 °C) through 5-step cooling/heating cycles. This temperature range is particularly relevant for outdoor lighting applications, where the QLEDs are required to operate at brightness levels exceeding 10,000 cd/m^2^. Our devices showed thermal stability, with minimal standard deviation in their performance parameters. The device efficiency parameters almost return to their initial values once the temperature returns to room temperature at each step. This behavior is correlated to the changes in charge transport characteristics and the induced radiative/non-radiative exciton relaxation dynamics at different temperatures. The improved carrier mobility at elevated temperatures was also confirmed by the results of the unipolar EOD and HOD. The experiments were repeated for six different devices with consistent and reproducible results.

Due to their aerobic stability, our solution-processed ultrabright QLEDs are cost effective and feasible to large-scale fabrication methods, such as inkjet and roll-to-roll printing making them suitable for industrial applications, such as indoor and outdoor lighting. Moreover, thermal stability of our QLEDs proves their suitability to operate at harsh weather conditions.

## Methods

### Materials

Cadmium oxide (CdO, 99.99%, trace metals), trioctylphosphine (TOP, 90% technical grade), sulfur (S, 99.98%), 1-octadecene (ODE, 90%, technical grade), iso-octane (99.7%, HPLC grade), 1-octanethiol (> 98.5%), trimethylammonium chloride (TMACl, > 98%), potassium hydroxide (KOH, 99.99%), dimethyl sulfoxide (DMSO, > 99.9%) magnesium acetate tetrahydrate (99%), zinc acetate dihydrate (> 98%), lithium acetate (99.95%) and 1-butanol (anhydrous, 99.8%) were purchased from Sigma-Aldrich. Zinc acetate anhydrous (+ 99.9%) and ethyl acetate (> 99.5%, ACS certified) were purchased from Thermoscientific. Selenium (Se, 99.999%, metals basis) and oleic acid (90%, technical grade) were purchased from Alfa-Aesar. Octane (+ 99%, extra pure) was purchased from Acros Organics. All the reagents were used as received. Poly(ethylene dioxythiophene): polystyrene sulfonate (PEDOT: PSS) was purchased from Ossila. Poly[(9,9-dioctylfluorenyl-2,7-diyl)-co-(4,4’-(N-(p-butylphenyl)) diphenylamine)] (TFB) was purchased from American Dye Source. Polyvinylpyrrolidone (PVP10) with an average molecular weight of 10,000 was purchased from Sigma-Aldrich. Patterned ITO-glass substrates with 15 Ω resistance and 25.4 mm × 25.4 mm × 0.7 mm were purchased from Luminescence Technology Corp.

### Synthesis of ZnCdSe core QDs

CdO, zinc acetate, oleic acid and ODE were mixed with each other and degassed at 130℃. The temperature was raised to 300℃ and Se precursor in TOP was injected into the reaction flask. After core growth, the TOPSe precursor solution mixed with ODE was injected into the reaction flask (formation of ZnSe shell). Then, Se-S precursor was injected into the reaction. The sulfur solution (in TOP and ODE (1:3 vol%)), was added to octanethiol and injected into the flask. Then, the reaction was cooled down to 110℃. The QDs were washed with reagent alcohol/acetonitrile, and redispersed in hexane. After the final step of the precipitation, the QDs were dispersed in octane.

### Synthesis of Li-Mg-doped ZnO NPs

TMAH was synthesized by dissolving 2.2 g of TMACl in 14 ml of reagent alcohol, and 1.1 g of KOH in 16 ml of reagent alcohol, separately. After the complete dissolving, the KOH and TMACl solutions were mixed and centrifuged at 5000 rpm for 3 min. The resulting TMAH was filtered using 0.22 μm PTFE syringe filters, while the KCl solid part was separated. Next, 10%Li:10%Mg:ZnO (LMZO) was synthesized by solution-precipitation method with some modifications^51^. 8 mmol of zinc acetate dihydrate, was mixed with 1 mmol of magnesium acetate dihydrate, and 1 mmol of lithium acetate hydrate, and dissolved in 25 ml of dimethyl sulfoxide (DMSO). Then the temperature of the solution was reduced to below 2 °C and 21 ml of TMAH was added dropwise, and the solution was stirred for 2 h at T < 2 °C. The nanoparticles were then washed twice with ethyl acetate and redispersed in butanol.

### QLED device fabrication

All the device spin-coating processes were done in the open air, at room temperature with a moisture level of 11–15% in winter, and 25–35% in summer. The ITO-glass substrates were sonicated in a bath of detergent and DI water, acetone, and IPA. The substrate surfaces were then treated with a UV-Ozone lamp for 15 min. The PEDOT:PSS HIL was spin-coated at 5000 rpm for 40 s, and annealed at 130 °C for 20 min. An 8 mg/ml TFB solution was prepared in toluene and spin-coated at 3000 rpm for 40 s, the film was annealed at 110 °C for 20 min. ZnCdSe/ZnSe/ZnSeS/ZnS QDs were spin-coated at 3000 rpm for 35 s, and annealed at 80 °C for 15 min. 5wt% PVP was dissolved in 30 mg/ml LMZO and spin-coated at 3000 rpm for 35 s, then annealed at 80 °C for 20 min. Finally, a 50 nm-thick Al and a 120 nm-thick Ag were deposited as the cathode by thermal evaporation. The devices were then encapsulated with a UV-curable epoxy.

### EOD fabrication

After cleaning the ITO substrates, LMZO doped with PVP was spin-coated at 3000 rpm for 35 s, then the film was annealed at 80 °C for 20 min. QDs were spin-coated at 3000 rpm for 35 s, and annealed at 80 °C for 15 min. The second layer of the doped ZnO NPs was spin-coated on top of the QDs at 3000 rpm, for 35 s, and annealed at 80 °C for 20 min. The Al and Ag were deposited by thermal evaporation. The devices were then encapsulated with UV-curable epoxy.

#### **HOD fabrication**

PEDOT:PSS HIL was spin-coated at 5000 rpm for 40 s, and annealed at 130 °C. An 8 mg/ml TFB solution was prepared in toluene and spin-coated at 3000 rpm for 40 s, the film was annealed at 110 °C for 20 min. QDs were spin-coated at 3000 rpm for 35 s, and annealed at 80 °C for 15 min. A 5 mg/ml of PVK solution was in 1,4-dioxane and spin-coated at 4000 rpm for 40 s, then the film was annealed at 100 °C for 20 min. A 5 mg/ml TFB solution was prepared in p-xylene, spin-coated at 4000 rpm for 40 s and annealed at 100 °C for 20 min. The samples were then transferred to the thermal evaporation chamber, while 8 nm of MoO_3_, and 200 nm of Ag were deposited by thermal evaporation. The devices were then encapsulated with UV-curable epoxy.

### Characterizations

#### Materials

The PL spectrum of the QDs was characterized using a StellarNet Miniature UV–Vis spectrometer. The absorption spectrum of the QDs and LMZO NPs were measured using Cary 60 UV–Vis spectrometer from Agilent Technologies. The QLED layer stack structure and the thickness of each layer was characterized by TEM-cross sectional image. TEM and S/TEM/EDX analysis was performed on a JEOL ARM 200cf microscope, equipped with a cold-field emitter and probe Cs corrector and operate at 200 kV acceleration voltage.

#### Devices

The EL parameters were measured using a StellarNet Miniature UV–Vis spectrometer, and the QLED performance was measured by a Si-photodetector connected to a Keithly 2612B source meter, while the devices were pre-biased at 7 V before the testing, with a DC power supply. The device was run and the data was collected using a Python code, by which the voltage was swept from − 2 to 12 V and EQE_max_, L_max_, J, V, PE_max_ and LE_max_ were calculated and collected using the QDs EL parameters.

#### Temperature-dependent PL, EL, QLED, EOD, and HOD testing

QLEDs were put in contact with a TEC1-12715 0-15V heat sink thermoelectric Peltier cooler using a piece of a 0.5 mm thermally conductive pad, from Thermal Right Co. The Peltier plate was connected to the corresponding poles of a DC power supply. By applying voltage, the Peltier was heated resulting in a rise in the QLED temperature. The surface temperature of the device was carefully monitored using a FLIR E8-XT infrared thermal camera, till it reached 85 °C. The QLED device was then operated, and the performance parameters were collected using a Si photodiode. For low-temperature testing, the connections of the positive and negative poles were switched, such that the surface of the Peltier cooled down to − 10 °C. In our experiments, the low-temperature limit (− 10 °C) was imposed by the limitations of the cooling capacity of the Peltier plate. Moreover, the upper temperature (85 °C) was limited by the thermal degradation of the UV-curable epoxy. Adopting a non-contact encapsulation solution with the epoxy resin could make it possible to perform the measurement at higher temperatures.

### Supplementary Information

Supplementary Information.

## Data Availability

The datasets used and/or analyzed during the current study available from the corresponding author on reasonable request.
