# Supplementary material for: An investigation on the cyclic temperature-dependent performance behaviors of ultrabright air-stable QLEDs
Source: Sci Rep. 2023 Aug 5;13:12713. doi: 10.1038/s41598-023-39952-3 (PMC10404233; doi:10.1038/s41598-023-39952-3)
Supplement: Supplementary file 1 — Supplementary Information. [file 41598_2023_39952_MOESM1_ESM.docx]

**Supplementary Information for An Investigation on the Cyclic Temperature-Dependent Performance Behaviors of Ultrabright Air-Stable QLEDs**

Saeedeh Mokarian Zanjani, Sadra Sadeghi, Afshin Shahalizad, Majid Pahlevani^*^

Dr. S. Mokarian Zanjani, Dr. S. Sadeghi, Prof. M. Pahlevani*

Department of Electrical and Computer Engineering, Queen’s University, Kingston, Ontario, K7L 3N6, Canada

Dr. A. Shahalizad*

Genoptic LED Inc., Calgary, Alberta, T2C 5C3, Canada

^*^E-mail : [majid.pahlevani@queensu.ca](mailto:majid.pahlevani@queensu.ca)

**Accelerated lifetime measurement for spin-coated QLED**

The accelerated lifetime measurement was carried out at L_0_= 100000, 50000, and 10000 cd/m^2^. The half-lifetime (T_50_) at each initial brightness level was measured, and based on the equation below:

$T_{50,L}=T_{50,L_{0}}\times\left( \frac{L_{0}}{L} \right)^{n}$ eq. 1

Log T_50,L_=Log T_50,L0_ + n Log ($\frac{L_{0}}{L}$) eq. 2

By having 3 points and sketching them in logarithmic scale, the slope of the linear fit, shows the acceleration factor (n), which is 1.6 in our devices.


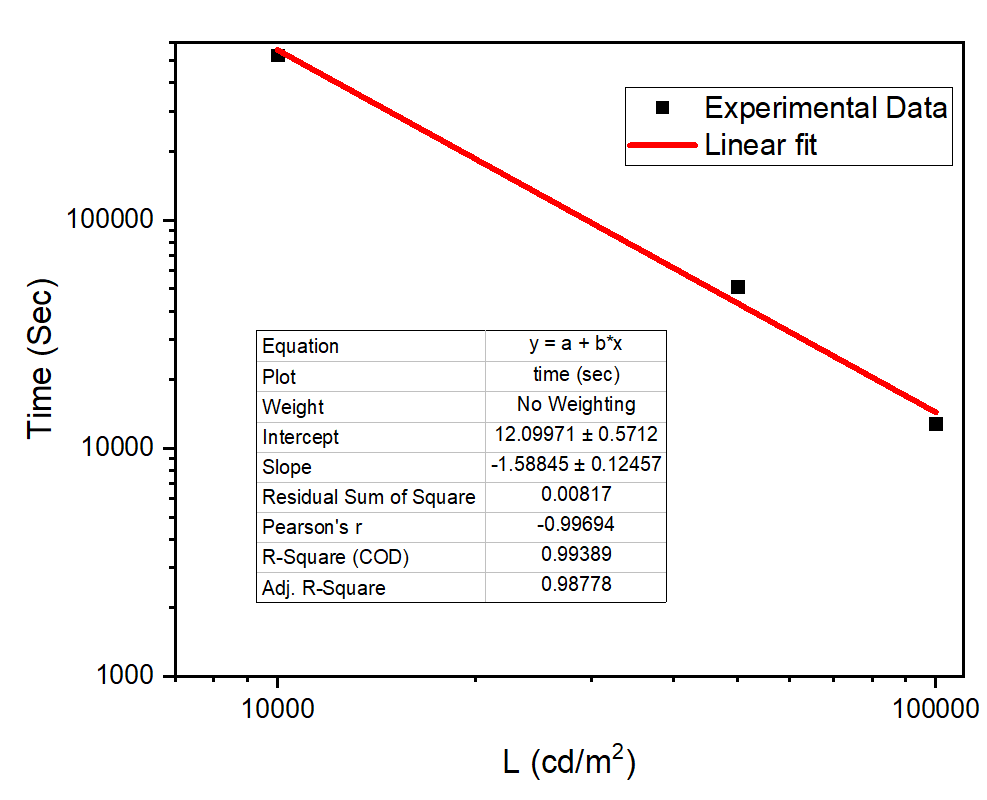


The experimental data of the half-lifetime measurements at each brightness level are plotted bellow. From the data of which, the values of T_70_, and T_95_ can be achieved.


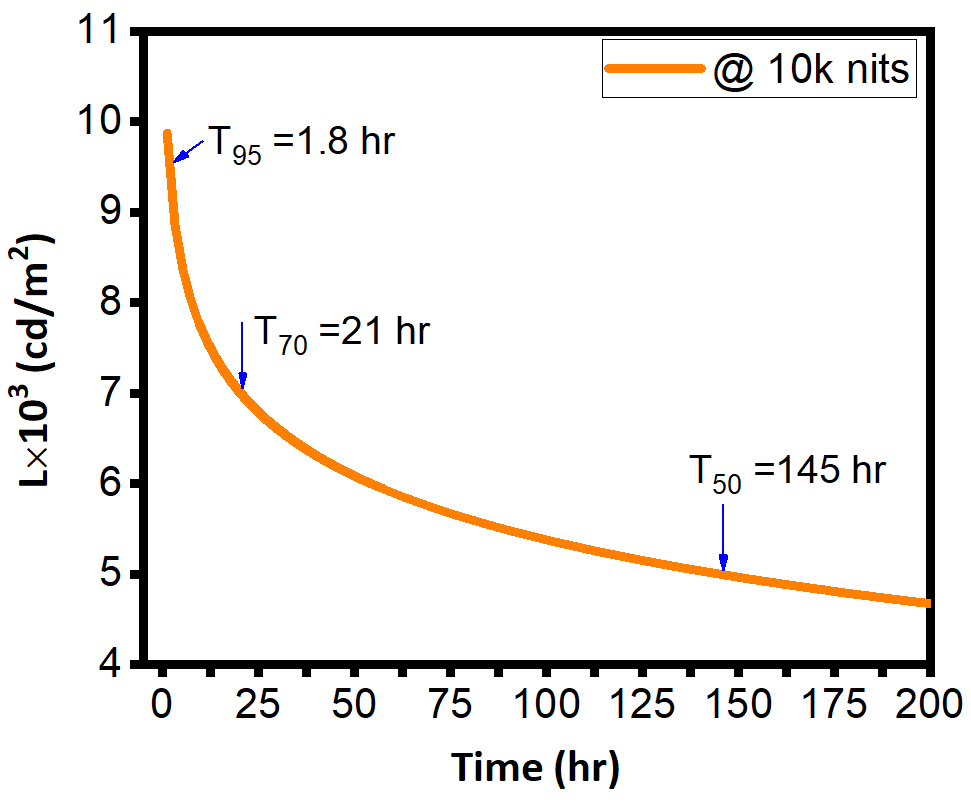

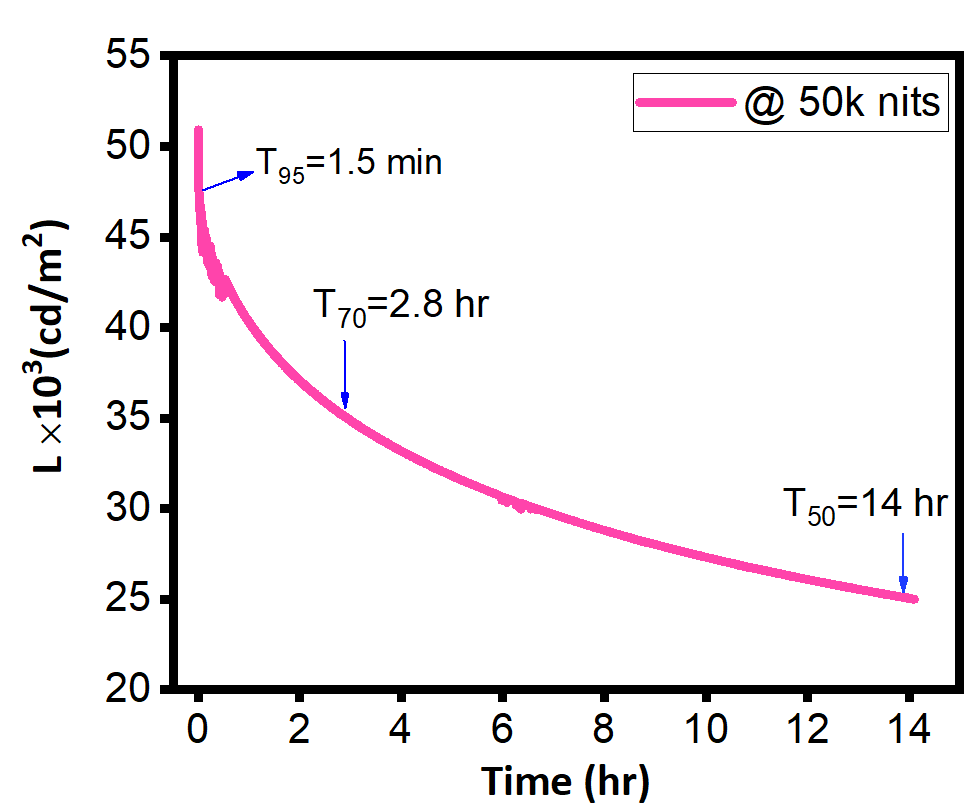


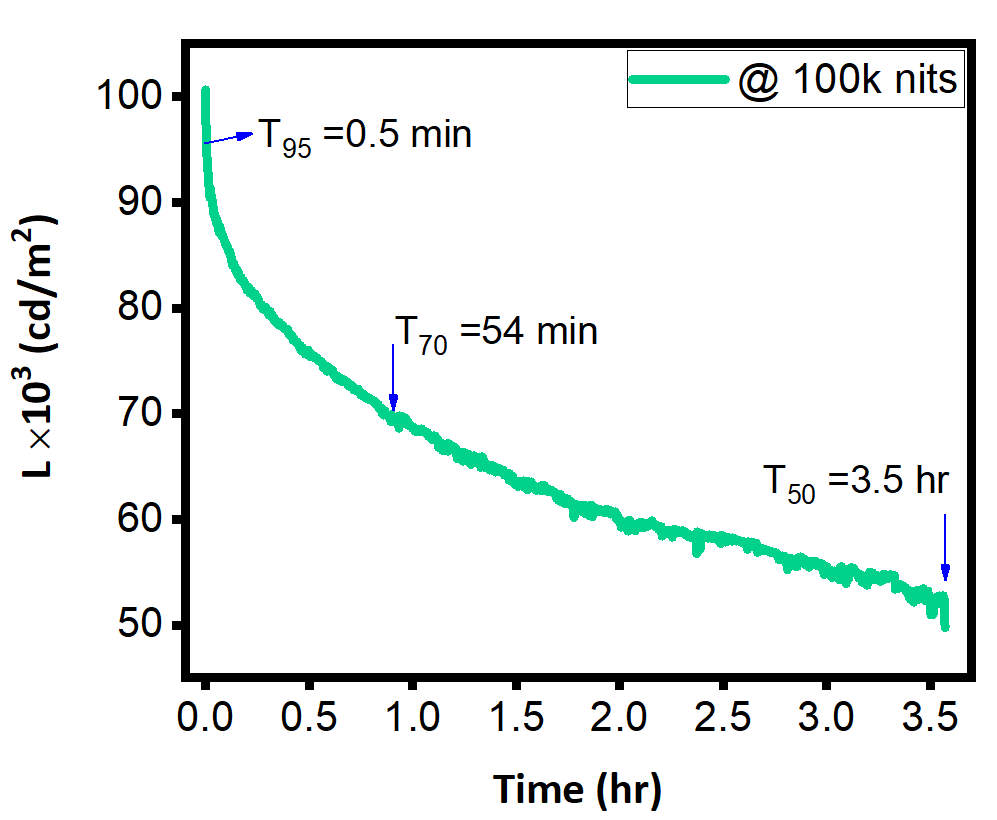


From the equation 1, and n=1.6 the values of T_50_, T_70_, and T_95_ for various brightness levels are calculated. The results are summarized in the table below:

| **L_0_ (cd/m^2^)** | **T_50_ (hr)** | **T_70_ (hr)** | **T_95_ (hr)** |
| --- | --- | --- | --- |
| 100 | 229810 | 33283 | 2853 |
| 1000 | 5800 | 836 | ~ 72 |
| 2000 | 1904 | 276 | ~ 24 |
| **10000** | **145** | **21** | **1.8** |
| **50000** | **14** | **2.8** | **1.5 min** |
| **100000** | **3.5** | **54 min** | **0.5 min** |
